# Mealtime situations in nursing homes from the residents’ perspective – an integrative review

**DOI:** 10.1186/s12877-025-05753-8

**Published:** 2025-02-17

**Authors:** Carina Werkander Harstäde, Stefan Andersson, Erika Lagerbielke, Anna Sandgren, Kristiina Heikkilä

**Affiliations:** 1 Department of Health and Caring Sciences, Linnaeus University, Kalmar, Växjö, Sweden; 2 Department of Music and Art, Linnaeus University, Växjö, Sweden; 3 Center for Collaborative Palliative Care, Department of Health and Caring Sciences, Linnaeus University, Kalmar, Växjö, Sweden

**Keywords:** Care homes, Five aspect meal model, Integrative review, Mealtime, Older people, Older people’s perspectives

## Abstract

**Background:**

When moving to a nursing home, the new resident also meets a new kind of culture, including communal, shared meals. Gaining greater insight into the resident experience of mealtimes in nursing homes is essential to identify the meaning that mealtime situations have and highlight the potential barriers and facilitators to the implementation of mealtime situations that create wellbeing for residents. The aim of this integrative review was therefore to shed light on mealtime situations in nursing homes from the residents’ perspective.

**Methods:**

The literature search was performed using a combination of MeSH keywords and free text terms in ASSIA, CINAHL, PsycInfo, PubMed, and Web of Science. After scrutinizing the articles and quality checks, 13 articles were chosen. The analysis was performed following Whittemore and Knafl’s instructions for integrative reviews.

**Results:**

The experiences of the mealtime situations were partly connected to residents’ personhood and partly to the nursing home’s organization of the mealtimes. Three main categories emerged: mealtimes as a source of well-being in nursing home life, mealtimes (re)creating continuity in life and preserving identity, and mealtimes as a balancing act between autonomy and need of support. Residents in nursing homes want the possibility to choose both what to eat, with whom they will eat and where they eat. The mealtimes in nursing homes and how they are constructed have an important role in residents’ feelings of having control over their life situation and can also strengthen residents’ identity and autonomy.

**Conclusion:**

Staff needs to be aware of the meaning of mealtimes to promote person-centered care in regard to meals. Further research is needed to explore how different mealtime situations have an impact on nursing home residents’ lives as well as on the care the staff gives.

## Background

Relocation to a nursing home is recognized often as an overwhelming change for older people [1]. When they move to institutional old age care (hereafter called nursing homes), their individual way of life may be challenged by the organization and rules of the nursing home, and they live together with people they have not chosen to live with [2, 3]. They have to leave part of their individual lifestyle and be part of the nursing home life [3]. Their individual choices may become more or less limited as they enter a collective lifestyle to which they need to adapt, but on the other hand, they are gaining care and a sense of safety [2, 4].

The construction of old age care and nursing homes (and the different designations for nursing homes) vary between different countries. Common is however, the possibility to get nursing and medical care when needed and that the care includes meal services. In most nursing homes, these meals are served at predetermined times and are part of the collective experience [5]. Mealtimes are thus social situations, where the meal is shared with the other residents in a dining room. The residents become, in a sense, unvoluntary guests at mealtimes, where the outer frame is partly decided by others: organization, staff and other residents [2, 5]. This may be the reason why mealtime environments were associated with lower satisfaction of dignity and wellbeing. Residents who did not find the mealtime to be a pleasant time of day had high odds of being dissatisfied with dignity and well‐being [6].

Mealtimes in nursing homes consist of the *product* (food and beverages available), the *room* (the local where the meal is enjoyed), and *the meeting* (encounters with those participating in the mealtime). These three aspects frame the *atmosphere* in which the meal is consumed, and all four aspects depend on the *management control system* in which the mealtimes are organized, according to the Five Aspects of Mealtime Model (FAMM) [7]. In nursing homes, the product (food) is often prepared by professional cooks/chefs, based on nutritional guidelines, and eaten in for that purpose designed dining rooms (room), where the residents are expected to socialize with other residents and/or staff (meeting). The mealtime atmosphere depends not only on these aspects but also on the management control system of the scheduled mealtimes and whether the residents are able to choose their seating and table mates [8]. This model will be discussed later in this paper.

Although mealtimes in nursing homes are planned for the collective needs and wishes, with limited possibilities to personal individuality and thus, risk to lack person-centredness [2]. Mealtimes can also be described as being together, creating a personal feeling of pleasure and company [9]. Communal dining experience, can thus be seen as a significant social event contributing to the quality of life preserving social connections [10]. Therefore, the mealtime situations are vital, as how the residents experience them influences both their well-being and their food intake [11–13].

A review of mealtimes in hospital settings and rehabilitation units showed that mealtimes in caring settings should be viewed as high priority where staff should be present to encourage sufficient food intake and that communal dining rooms has a potential to increase food intake and provide a social environment for eating [14]. Gaining greater insight into the residents’ experience is essential to identify ways of improving mealtime situations and care provision during these and can highlight the potential barriers and facilitators to the implementation of future interventions. We have found only one earlier review of mealtimes in nursing homes [15] where the residents’ experiences are described. In that review mealtimes were, however, studied both from the residents’ and staff’s perspective. Thus, there is still a need for a review that concentrates on residents’ views, to create deeper knowledge of their views and experiences to guide future development of mealtimes in nursing homes. The aim of this integrative review was therefore to shed light on mealtime situations in nursing homes from the residents’ perspective.

## Materials & methods

### Design

An integrative review according to Whittemore and Knafl [16] was performed. This allowed the inclusion of diverse qualitative, quantitative, and mixed methodology studies. The review process involved identifying, critiquing, and synthesising the literature.

### Ethics

As an integrative review, the study does not need ethical approval according to Swedish law [17]. The studies included in the review all had ethical approval or clear ethical statements.

### Literature search, eligibility criteria and implementation

The literature search was performed by the first and last authors. The choice of databases (ASSIA, CINAHL, PsycInfo, PubMed, and Web of Science) was based on communication with and information from librarians at the university library in order to reach a wide range of caring- and health science research. Database searches were performed using a combination of controlled vocabulary thesaurus from each database together with free text terms to find relevant keywords. The specific search keywords were combined using Boolean terms in different ways (Table 1) to both broaden and narrow the searches. The limitations used were adjusted to each database: peer-reviewed, original research article, full text available, in English, and published 2012 or later. The searches were conducted from November—December 2021, with a complementary search for 2022 in January 2023, and one paper was added after manual search. Reasons for keeping the timeframe to ten years was that a longer timespan was considered relevant to gather rich data for the analysis.

**Table 1:** Keywords search strategy

Old* adult* OR Old* people OR resident* AND Experience* OR Lived experience* OR Perception* AND Long-term/longterm care OR Nursing home* OR Residential care AND Mealtime* OR Mealtime behav* OR Mealtime situation OR Meal environment* OR Eating	

**Fig. 1:**
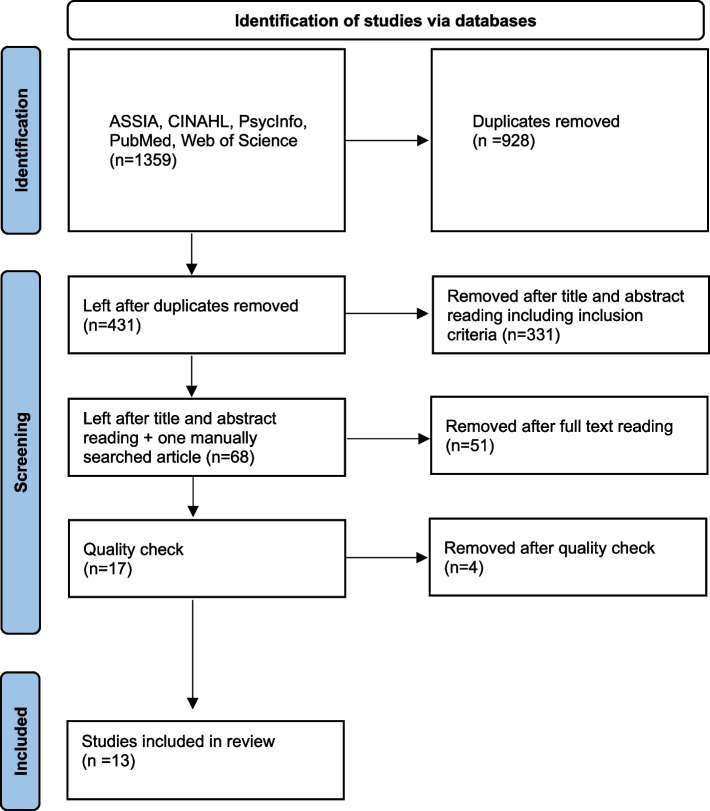
PRISMA flowchart

By narrowing the searches, a title list was created, resulting in 1359 titles read. Here, duplicates were removed. This led to 431 articles that went further to abstract reading where the inclusion criteria were as follows: the study should report residents’ perspectives; the context should be the nursing home; and the study should be about mealtime situations. This narrowed the list down to 67 articles that were read in full text. This reading showed that 51 articles had not the residents’ perspective but the next-of-kins’, staff’s, or researchers’ perspective. Here the article that was manually searched was added. The remaining 17 articles went through a quality appraisal, and 13 articles were included in the review. See PRISMA flowchart (Fig. 1).

### Quality appraisal

Quality appraisal was performed to assess the methodological quality in each study and to determine if and how much the study addressed the possibility of bias in its design, conduct and analysis. As mentioned, 16 studies went through quality appraisal, which was performed by the first and last author using the JBI Critical Appraisal Tools [18]. These tools focus on what counts as evidence in a study and what method is used to synthesize this evidence, something that was considered important to appraise quality. The versions for qualitative studies, quantitative studies and prevalence studies were used. According to the Swedish Agency for Health Technology Assessment and Assessment of Social Services [19] different questions in assessment tools can have different importance and affect the final assessment in different ways, depending on the area being investigated. In this study decisions were made that to receive a moderate degree of quality, 65% of the questions in the critical appraisal tool should be answered by yes. High quality, 85%. Four studies did not reach a moderate level and were excluded. After the appraisal, 13 studies were included in the review; of these, nine studies were qualitative, three used mixed methods, and one was quantitative (see Table 2).

**Table 2:** Included articles

Article	Settings and participants	Aim	Methods	Findings	Quality appraisal
Adams, K, Anderson, JB, Archuleta, M, Kudin, JS (2013). Defining skilled nursing facility residents’ dining style preferences. *Journal of Nutrition in Gerontology and Geriatrics*, 32 (3): 213–232 USA	Three skilled nursing facilities (SNF) in Texas, USA 104 residents who met the cognition criteria and consumed facility-provided food	To determine residents’ home dining practices, to define residents’ desired dining style practices in the SNF, and to determine the relationship between home dining practices and dining preferences in SNF	Standardized interviews using the Resident Dining Style Preferences Survey Data were analysed to determine the degree to which home practices determine SNF dining preferences	The majority of the participants wanted hot, home cooked meals served in the dining room. They wanted to be seated at the table with friends and neighbors and served on everyday plates in a quiet atmosphere, with food served restaurant or table service style. Length of stay and generational group were not significant predictors of dining style preferences	High
Buckinx F, Reginster JY, Morelle A, Paquot N, Labeye N, Locquet M, Adam SB (2017). Influence of environmental factors on food intake among nursing home residents: a survey combined with a video approach, *Clin Interv Aging*. 4 (12):1055–1064 Belgium	Nine different nursing homes in Belgum 88 residents, 18 experts and 45 nonexperts¨	To assess, by means of an original video approach, the influence of the environment on food intake in nursing homes	A random sample of residents answered a self-administered questionnaire related to different indicators (i.e., noise, space, comfort, light, odors, perceived satisfaction of meals, taste of meals, presentation of meals, service and setting) Two separate panels (expert in geriatrics, non expert in geriatrics) answered a questionnaire on their perception of the environment after having watched a video of the lunch in each nursing home The food intake of the residents was measured by food-weighting	The perception of the quantity of food served by the residents is the only single factor associated with food consumption (*P* = 0.003) On the other hand, experts and nonexperts did not perceive any environmental factor that seems to be significantly associated with residents’ food intake	High
Girard, A, El Mabchour, A. (2019). Meal context and food offering. International *Journal of Migration, Health, and Social Care*; 15 (3): 226–246 Canada	Care facilities in Quebec, Canada 26 non-Quebec-born residents 24 of their family members 51 frontline care staff	To gain a better understanding of the meal context and the food offering in Quebec public nursing homes for nonautonomous seniors, particularly with respect to first generation immigrants	Focused ethnography Interviews with 3 groups: 26 non-Quebec-born residents 24 of their family members 51 frontline care staff Structured nonparticipative observations were made in facilities	First-generation immigrants, independent of how long ago they arrived in Quebec, seldom adapted the Quebecian food offerings. Resident’s appetite for food offer was a problem for reasons related primarily to food quality, mealtime schedules, medication intake, physical and mental condition, and adaptation to institutional life. Family and friends often brought in food	High
Kenkmann, A, Hooper, L (2012). The restaurant within the home: experiences of a restaurant-style dining provision in residential homes for older people. *Quality in Ageing and Older Adults,*13 (2): 98–110 United Kingdom	Four medium-sized (approximately 35 residents) local authority care homes in Norfolk, UK 16 residents 32 staff	To explore the experiences of residents and staff with new restaurant-style meal provision in four residential care homes in Norfolk, England	Observations of meal and drink provision during a whole day in each home Unstructured individual interviews with residents and staff Content analysis	Although older care home residents enjoyed the restaurant experience, they valued stable table companions more highly than flexibility Residents appreciated attractive surroundings, good food and their possibility to make choices. However, in some circumstances and for frailer residents, choices were more limited While the central restaurant was valued for the main meal some residents preferred smaller ‘‘family-type’’ dining for other meals Available space and the dining-room’s location contributed to the success of the restaurant-style provision	Moderate
Mahadevan, M, Hartwell, HJ, Feldman, CH, Ruzsilla, JA, Raines, ER (2014). Assisted‐living elderly and the mealtime experience. *Journal of Human Nutrition and Dietetics,* 27 (2): 152–161 USA	Four assisted living facilities in Monclair, NJ, USA A convenience sample of 38 men and women, aged ≥ 65 years	To critically evaluate the food voice of elderly residents in an assisted-living environment	One focus group discussion in each of the facilities with 8–10 participants aiming to capture the feelings, emotions and viewpoints of assisted-living residents Content analysis	Participants ability to make healthy food choices, socialise, interact with staff, friends and family members and enjoy a tasty meal in a warm and inviting dining environment to provide a dignity that is unmatched by other services	Moderate
Milte, R, Ratcliffe, J, (…), Crotty, M (2018). Taste, choice and timing: Investigating resident and carer preferences for meals in aged care homes. *Nursing & Health Sciences,* 20 (1):116–124 Australia	Three aged care homes in Australia 43 residents 78 of their family members	To undertake a detailed analysis of the preferences for how food and the dining experience are provided within aged care homes from the perspective of residents (without cognitive impairment or with mild cognitive impairment) and informal carers predominantly family members of residents experiencing moderate and severe cognitive impairment), including an analysis of their WTP for these preferences	A discrete choice experiment questionnaire administered via interview. A DCE questionnaire comprising of four mains sections administered by a trained interviewer	Participant preferences were influenced by food taste, choice in relation to serving size, timing of meal selection, visual appeal, and additional cost The study found that respondents were willing to pay a premium to receive food that met their expectations of taste, and for a high level of control over serving sizes	High
Okkels SL, Saxosen M, Bügel S, Olsen A, Klausen TW, Beck AM (2018). Acceptance of texture-modified in-between-meals among old adults with dysphagi, *Clin Nutr ESPEN*. 25:126–132 Denmark	Three Danish nursing homes 30 old adults ≥ 70 years suffering from dysphagia	To identify the most liked in-between-meals for old adults based on flavour and describe the basic sensory properties of these in-between-meals	Participants assessed 20 texture modified in-between-meals based on their flavour and appearance on a 3 point hedonic scale	When participants were asked to assign liking based on flavour, the most liked in-between-meals were frozen, cold and sweet (vanilla ice cream, strawberry parfait and panna cotta). These meals were among the in-between-meals richest in fat and energy Liking based on flavour and appearance was equal in 18 out of 20 samples	High
Okkels, SL, Dybdal, DR, Beck, AM, Bügel, S, Klausen, TW et al.(2019). An investigation of main meal preferences in nursing home residents. *Journal of Sensory Studies*, 34 (4): 1–10 Denmark	One Danish nursing home 60 residents 29 residents participated. They were ≥ 70 years, had no swallowing problems	To investigate basic sensory customization factors of most preferred main meals in nursing home residents	Forty popular Danish meals Tested. Thirty of the meals contained meat or fish and the rest were plant-based Residents evaluated liking of appearance, flavour, and texture on a 5-point liking scale in eight tasting sessions with five food samples per session	In meals containing pork, meal appearance scores were significantly higher than the meals containing beef (*p* = .039). Meals containing beef scored significantly higher on flavour than the plant-based meals (*p* = .025). Meals containing fish scored higher on texture than meals containing beef (*p* = .003), and plant-based meals (*p* = .005)	High
Palacios-Ceña D, Losa-Iglesias ME, Cachón-Pérez JM, Gómez-Pérez D, Gómez-Calero C, Fernández-de-las-Peñas C (2013). Is the mealtime experience in nursing homes understood? A qualitative study. *Geriatrics & Gerontogyl International,* 13(2):482–9 Spain	Nonprofit nursing homes in Madrid, Spain 26 residents who were ≥ 60 years (mean age 83 years) with no cognitive impairement	To explore the significance of the mealtime experience among residents of nursing homes in Spain	A qualitative phenomenological approach Unstructured and semistructured interviews	Three main themes that describe the significance of meals in nursing homes: (i) timing of the meals – mealtimes serve as a point of reference for organizing activities in the nursing home and orient the residents during the day; (ii) table allocation – table allocation depends on the judgment of the personnel, the behavior of each resident and on the input from the residents that use a table; and (iii) the meals themselves – food is experienced as a privilege, as a sign of autonomy and normality, and as an indicator of personal identity	Moderate
Philpin, S, Merrell, J, Warring, J, Hobby, D, Gregory, V (2014). Memories, identity and homeliness: The social construction of mealtimes in residential care homes in South Wales. *Ageing & Society*, 34 (5): 753–789 United Kingdom	Two different type of residential care settings in Wales, UK 16 residents 10 informal carers 15 staff members 4 managers	To investigate factors influencing the nutritional care provided to residents in two different types of residential care settings	Focus group interviews with the four group of participants observations of food preparation and mealtimes throughout the day analysis of appropriate documents Thematic analysis	Residents’ experiences and understandings of mealtimes were shaped by the interlinked physical and sociocultural environment of their lives. The physical elements of the environment included the homes’ geographical locations and their physical layouts, both of which underpinned residents’ experiences and their sense of community and identity. Thus, these physical features link to sociocultural elements of the environment, which are complex and include people’s sociocultural backgrounds, their family experiences and memories, and their sense of community and identity; all of which inform their understanding of mealtimes	High
Sjögren Forss K, Nilsson J, Borglin G. (2018). Registered nurses’ and older people’s experiences of participation in nutritional care in nursing homes: a descriptive qualitative study. *BMC Nurs*., 17(19): 1–13 Sweden	Six Swedish nursing homes 4 older persons (mean age 85,7 years 8 registered nurses	To illuminate the experience of participating in nutritional care from the perspectives of older people and registered nurses. to illuminate the latter’s experience of nutritional care per se	Semitructured interviews Content analysis	Three themes: ‘participation in nutritional care equals information’, ‘nutritional care out of remit and competence’ and ‘nutritional care more than just choosing a flavour’ They illuminated the experience of participation in nutritional care from the perspective of older people and RNs, and the latter’s experience of nutritional care in particular per se	High
Wang, D, Everett, B, Brunero, S, Northall, T, Villarosa, AR, Salomonsson, Y. Perspectives of residents and staff regarding food choice in residential aged care: A qualitative study. Journal of Clinical Nursing; 2019; 29:626–637 Australia	Two nursing homes 7 residents 7 staff	To explore the experiences of food choice and meal services in residential aged care facilities and its impact on autonomy, self-determination, and quality of life from the perspective of both residents and staff	In-depth, semi-structured interviews Thematic analysis	Three main themes were identified: (1) catering for the masses; (2) organisational barriers to providing choice; and (3) food impacts well-being	Moderate
Watkins R, Goodwin VA, Abbott RA, Hall A, Tarrant M (2017). Exploring residents’ experiences of mealtimes in care homes: A qualitative interview study, *BMC Geriatr,* 11;17(1):141 United Kingdom	Four care homes in the South West of UK 11 residents	To gain an insight into the dining experiences and explore some of the issues that may impact on residents’ enjoyment of meals, and resulting health and wellbeing	Semistructured interviews Researcher observations of mealtimes Thematic analysis	The dining experience was a focal point for participants’ broader experiences of residing in a care home Three themes pertaining to residents’ experiences were identified: (1) Emotional and psychological connections with other residents; (2) managing competing interests with limited resources; and (3) familiarity and routine	High

### Data analysis

Based on Whittemore and Knafl [16], the data analysis was performed based on data reduction, data display, data comparison, conclusion drawing, and verification.

Data reduction comprised determining a classification system to manage the data from diverse methodologies by dividing the studies into subgroups to facilitate analysis. It also involved extracting, coding, and classifying data from each study, that is, author, publication date, country of origin, setting and participants, aim, methods and findings (Table 2).

In the data display the extracted data that answered the aim of shedding light on mealtime situations in nursing homes from the residents’ perspective was converted into a display that assembled the data from the different sources. Post-it notes were used to develop the idea of patterns and relationships within and across the data and was thereby a starting point for interpretation.

The data comparison step involved an inductive comparison of the data, in order to identify patterns. The first and last author extracted data from the included studies. Data were displayed in spreadsheets to facilitate detecting patterns of similarities and differences.

Finally, conclusions were drawn where the interpretive effort from the description of patterns and relationships were moved to higher levels of abstraction. This conclusion drawing was repeatedly revised to include as much data as possible. It was also verified by the coauthors.

## Results

The results are based on 13 studies; United Kingdom (3), USA, Australia, Denmark (2), Belgium, Canada, Spain and Sweden (1). In each study, one to nine nursing homes were involved, and data collection was conducted via individual interviews, focus group interviews/discussions, and different questionnaires. These nursing homes were labelled skilled nursing facilities, nursing homes/facilities, care homes, assisted living facilities, aged care homes, or residential care settings, thus involving older residents with different levels of care needs. A total of 4 to 104 residents in each nursing home participated in the studies. For more information, see Table 2.

The analysis showed that the experiences of the mealtime situations were partly connected to residents’ personhood and partly to the nursing home’s organization of the mealtimes (see Table 3 for categories and subcategories).

**Table 3:** Overview of categories and subcategories

Categories	Subcategories
Mealtimes as a source of well-being in nursing home life	*Mealtimes as social hubs*
	*The role of the mealtime environment and organisation of meals*
Mealtimes (re)creating continuity in life and preserving identity	*The role of routines*
	*The role of food*
Mealtimes as a balancing act between autonomy and need of support	*Food choice as a possibility for the sense of autonomy and control*
	*The role of food restrictions in the sense of autonomy and control*

### Mealtimes as a source of well-being in nursing home life

Mealtimes have an important role in the life at the nursing home. They are the occasions where the residents can experience social belonging with the people surrounding them and being part of the nursing home life entity.

The participating residents in the reviewed studies lived in nursing homes with a large variety of organisation of the meals and dining room solutions. The place where the mealtime was served was closely associated with the organisation of mealtimes in the nursing homes, and different solutions had impact on the residents’ experiences and satisfaction with the choices. To be served tasty food in a nice environment was important.

#### Mealtimes as social hubs

Residents described mealtimes as social occasions that provided comfort, well-being, and familiarity and helped to decrease feelings of depression and loneliness [20, 21]. Mealtimes were regarded as social situations and residents’ expectations of the mealtimes were connected to meeting friends and to chat. Even the waiting time for the opening of the restaurant doors was regarded to be part of the “restaurant experience”, while meeting others and watching the things happening around. However, if the waiting time in restaurant-style dining was long, it irritated the residents [20].

To be able to choose one’s table mates in the dining room was thus important for the well-being of many residents, as they wanted to eat with their friends [20–22]. Some residents arrived in the dining room up to 40 min earlier just to be sure to get the seat they wanted [21]. The mealtimes could therefore be seen as opportunities to establish and maintain relationships with other residents. Interactions between tablemates could establish emotional and psychological connections. There was also a ‘shared understanding’ or ‘unspoken bond’ between the residents sitting together, which could be seen as a sense of community, something that made the residents feel emotionally and psychologically connected. The staff had an influential role by facilitating these relations by supporting conversation either by aiding suitable table groupings or by sitting down and having their meals together with the residents [20].

Even if the residents had, in theory, the freedom to choose where to sit, in practice the placement could depend on the functional ability of the residents, so that the staff could easily help them. Also, the newcomers’ first seating seemed to remain as permanent seating and therefore also forced the resident to socialize with those around the table, something that could diminish their own will. There were also residents who preferred to be on their own otherwise, but sitting together during the mealtimes gave them opportunities to build and maintain social relations [20].

In some nursing homes, the table setting was decided by the staff, and being placed at a ‘good’ table with ‘good’ table mates was a reward of ‘good’ behaviour. At best, this resulted in the resident being able to choose the table mates and even that the table mates had the veto-right for unwanted persons to sit at the table [23]. ‘Bad’ behaviour instead led to isolated sitting or removing the resident from the dining room [22, 23]. When the staff had chosen the table placement for the residents, it could also lead to anger from residents, and they could experience that they had no decision right in the matter [24].

Residents with limited communication skills, e.g., hearing loss, still desired to build relationships and a sense of community, which was important for their well-being. A lack of social contact could be present when residents sat at the same table because they were not talkative or because problems with hearing made it difficult to have a conversation. Some residents just accepted that, while other still desired to build relationships and a sense of community as it was important to their well-being. They tried to find ways to facilitate communication on their own or with help from staff [25].

There were also residents who preferred to be on their own otherwise, but sitting together during the mealtimes gave them opportunities to build and maintain social relations [20].

Mealtimes could also create feelings of isolation when residents longed for their own family [26], as one fourth of the residents missed their family members at the meals [21, 26]. This could lead to sadness and lack of appetite [26].

When not feeling up to socialize with others or being fairly indifferent where they sat, while eating, could lead residents to prefer to eat in their own rooms, sometimes due to being ashamed of their eating difficulties [22, 27]. Moreover, for some residents, the choice of table mates was not important [22]. The residents could also feel uncomfortable with the other persons sitting around the meal table due to them crying out loud, coughing, and spitting. It made them spend as little time as possible in the dining room or preferably sit by themselves [22, 27].

#### The role of the mealtime environment and organisation of meals

Residents preferred restaurant-style rather than “family-style” catering, served on plate instead of buffet style [20, 21]. Moreover, the residents wanted restaurant-style setting with menu choices [20, 26, 28]. They appreciated the restaurant experience and that tables were decorated making the meal a feast [22, 26]. Food choices in the menu were part of the restaurant feeling that made the dining room a more inviting place that increased the enjoyment of the meal [20]. A majority wanted three meals a day to be provided by the nursing home [21]. If the main meals lacked choices, even smaller options, such as having possibilities to choose breakfast or having possibilities to get coffee or tea throughout the day, were appreciated [22, 24]. Having no or few food choices triggered negative feelings about portion sizes and taste of food [28, 29].

The residents regarded the restaurant in nursing homes to differ from the restaurants in the community, as the adjustments to the residents’ physical needs made the spatial environment more institutional-like. When the dining room/restaurant had self-service facilities, the choices that were available functioned well for those who had no physical limitations, while those with disabilities such as dementia disease or blindness had to wait for the staff and the choices staff made [22].

Meal arrangements were lifted as an important component, such as presenting the meal as appealing to the eye [26]. Attractive table settings, chandeliers and knowledgeable staff were appreciated and made the mealtimes more memorable [30]. Residents appreciated coloured, everyday plates and regular glasses and napkins instead of bibs [21, 30]. If the dining room was adjacent to the kitchen, this enabled the residents to feel the smells of food, which they appreciated, and it stimulated their appetite [25]. When these attributes were missing, the spatial environment of the mealtime area was regarded more institution-like [30]. The residents also seemed to regard the serving of food as being the staff’s work, as waiters and waitresses [22].

The residents preferred a calm meal environment in nursing homes, even if they had at their earlier home watched TV or listened to radio during their meals [21]. However, whether the dining room was perceived as cozy, noisy, spacious, or brightly lit did not significantly change residents’ food consumption or food intake [30].

Some residents regarded small dining room areas to be more intimate and relaxed in the atmosphere. A large restaurant area was considered by some residents to be ‘crowded’, as it made it difficult for them to hear [22]. Additionally, messy and crowded plates led to loss of appetite [26].

### Mealtimes (re)creating continuity in life and preserving identity

Residents appreciated having routines around the mealtimes and being served well-known food as it helped them to feel comfortable and in charge of their life in the nursing home context. By this, the routines and food helped to mediate who they had been and how they were now, thus creating a continuity in their life.

#### The role of routines

Moving to a nursing home was an interruption in the residents’ life and lifestyle [20], but mealtimes could help them feel continuity in life and preserve their identity and a sense of identity and continuity [21, 25, 27]. Being able to sit and eat with one’s chosen table mates on a daily routine helped to create continuity in nursing home life, as the residents then knew who they were going to meet, and the table mates became friends [22, 25].

Residents seemed to find comfort in the routines of the nursing home by choosing the same breakfast every day or by choosing familiar food when such was available [20]. Residents’ wishes for mealtime schedules corresponded with nursing home practices [21], although too early dinners could cause dissatisfaction [29]. Scheduled mealtimes became reference points to the day’s activities and thus gave a sense of control [21, 23, 24, 28] and structure for the day [20]. Even celebrating special occasions, such as birthdays or national feasts with special meals and decorations, evoked memories of the past but also broke daily routines in a positive way [20], thus giving a sense of continuity in life.

Following the etiquette for mealtimes and the routines and rules of the nursing home for some residents’ meant continuity in life and social normality, leading to integration and possibility for self-esteem and for continuity in identity [23].

#### The role of food

Sensing food smells from the kitchen stimulated appetite but also provided links to earlier memories [25]. Some residents wanted to be involved in food preparations, while others did not [28]. Lacking opportunities to participate in meal preparation, from buying the groceries, cooking the food, serving it, and cleaning the table afterwards, was a loss of identity for several female residents, as they had been doing it for decades [23].

Several residents missed the smell and taste of their own cooking, which often included the traditional national food that underpinned their cultural identity and evoked memories [25, 28]. Therefore, the residents wanted traditional food [20, 27, 28, 31, 32] and thus the culturally different food of the nursing home, either due to its modernity [25] or by not being their culturally well-known food [27, 29, 31], was not regarded as a “proper meal” or tasty. A few widowed men also felt lost when confronted with the new food, as they did not know how to combine the different ingredients being served. because their wives had always been the ones deciding for them what to put on the plate and how to combine the food [26].

### Mealtimes as a balancing act between autonomy and need of support

The residents wanted to live an independent life and be in charge of their lives. Food and mealtimes were part of this strive. Therefore, being able to make choices was important even when health problems restricted the choices, and the residents strived for keeping control of their mealtimes.

#### Food choice as a possibility for the sense of autonomy and control

Being independent was about having the right to be involved in one’s own life and decision making during the meals. Individual choices were important for autonomy [20] and for sense of control [23]. The choices could concern a will to be part of the shaping of the mealtime environment and the menu, mealtime schedules and the table setting and table mates. Many persons were even prepared to pay extra to be able to get meal choices they preferred [23].

When the nursing home offered limited food choices, the independence and autonomy of the residents were restricted [24, 26]. Nevertheless, few residents complained about the food they got, although they felt suppressed, as they would feel ashamed of not being grateful for what they got [24]. Although not being a favorite, by time, residents also became familiar with the nursing home food and started to prefer that as their main choice [30]. If the staff seemed busy, it could limit the possibilities to choose as the residents did not ask for more food even if they wanted more [22, 29].

Residents also wanted to be able to choose what to eat and drink even if they had problems due to health risks [28]. Some residents, even when there were different food choices available, trusted the staff to choose the food for them, either because they trusted the staff’s choices to be good or because the residents had restricted sight or other physical limitations [22].

#### The role of food restrictions in the sense of autonomy and control

The food choices could also be restricted by the residents’ health condition [22, 24, 27, 29]. Residents stated that their personal health condition was an important factor determining what they wanted to eat and when [26]. Those residents who had food restrictions were well aware of their health concerns, as well as that different residents had different restrictions [22]. At the same time some residents wanted to be able to choose what to eat and drink even if that could cause problems due to health risks [26].

Several residents complained about the lack of information about what was good/not good for them to eat [24, 26]. Some residents also regarded the registered nurse as having the power to make food decisions over their heads [24]. Lacking knowledge diminished the possibility of having control over nutritional choices [26], thus, residents felt a loss of control over their lives and were passive receivers of nutritional care and information [24].

Residents with nutritional problems often lacked food choices. Although they experienced that the staff tried to accommodate their wishes, the lack of food choices diminished their sense of control [24]. Due to restrictions in diet the residents had to accept food they were forced to eat instead of eating with pleasure, something that created sadness [26]. Although residents wanted to eat what was good for them and they felt that they knew what food to eat, they felt restricted if the staff made the choices for them [26]. This could lead to anxiety, i.e., when having always been slim and now needing to gain weight, something that was understood as a negative consequence of the food offered [24].

When the healthy diet restricted them from eating what they liked, it led to a loss of control and autonomy. This could result in residents choosing not to follow the prescribed diet, as they felt that choosing by themselves was a way to have control over their lives [26].

The residents were also concerned about trying to balance their independence and being provided necessary support. Most of the residents accepted the loss of independence in favour of support [20] and felt that the staff supported them when needed, which was appreciated [28].

## Discussion

This review shows aspects related to mealtime situations from the nursing home residents’ perspective. The aspects that were noted as important for the residents in the mealtime experience included all the aspects of FAMM [5]. These aspects also co-operated in complex ways. What was appreciated by the residents was *a product* of tasty, well-known food that was suited for their functional and nutritional restrictions and also promoted the sense of continuity. This included the communal meals in a dining room shared with others, where the *meetings*, social encounters with other residents, and staff enabled the understanding of the etiquettes and routines of the mealtime and gave continuity for their identity. The meeting preferably took place in a restaurant-style *room* with few obstacles and everyday porcelain. The mealtimes should have a cozy, calm *atmosphere* and be organized so that the whole mealtime situation was easy to capture, and the care staff was a helping hand (*management control system*).

But the meaning of mealtimes is more complex than getting nourishment and company in a well-organised, ‘cozy’ room and atmosphere. Mealtimes situations in nursing homes are associated with thoughts of self-esteem, continuity in life, autonomy, and independence. Our results support Morgan’s results that mealtimes are central to well-being and involve physiological aspects as well as social interactions [33]. In our review, we found that mealtimes also had a meaning connected to the residents’ striving to be in charge of their lives. Therefore, personal identity was linked to the possibility of choosing what to eat and with whom [15].

In accordance with our review, residents regard individual choices and autonomy as crucial challenges in mealtime situations since nursing homes are often organized in an institutional way (cf. 15). Here, Harnett and Jönsson [4] take a more critical approach in interpreting residents’ choice of seatings. They ask whether this is an attempt to mark out personally owned, private space in a common room or as a case where residents are upholding the institutional order at the nursing home. In their observations, they found both the community trying to enforce the institutional frame upon the new residents and residents appearing to create personal space [4]. The residents’ wish to maintain autonomy can create tension with staff and organisations’ need to limit risks for the residents; therefore, the staff may want the residents to follow the nursing home rules to ensure the residents’ safety [34]. Thus, the staff role is connected to the complex power relationships in nursing homes. When the care staff decided where the residents should sit and with whom, they exercised power. This paternal power exercise seemed to be typical when the residents had food restrictions, as the residents were seldom informed of the usefulness of the diet and thus lacked the possibility to make independent decisions, although the aim was good.

Relations between residents, often brought into focus at mealtimes, show challenges in generating a social and tolerant atmosphere in nursing home settings where residents have different physical and emotional needs. The seating seemed to be a crucial question. Research shows that the “institutional meal script” was dominant in nursing homes as a way to label an established type of frame with distinct episodic content and role expectations within meals, where the staff regarded the institutional practices to be for the sake of having a functioning order in nursing homes. This institutional meal script provided normative expectations for residents’ eating performance [4]. Our results find the question even more complex; when the seating in the dining room was free, the residents had autonomy and could sit with their friends. This can also be connected to the importance of appropriation for residents in nursing homes to help to feel continuity in life when they routinely sit down in the same place in the dining room, as the sitting is a way to maintain daily routines and experiences of a personalized environment [34]. However, those who were not wanted as table mates due to their functional restrictions or due to their eating habits were left alone, with few possibilities to socialize with others. When the staff decided the sitting place, those residents were benefitted, as they could also have people to socialize with, although the staff’s “institutional script” diminished the residents’ autonomy.

Our results highlight how mealtimes helped the process of adjustment to institutional life through well-known food with familiar tastes and smells by celebrating national holidays and thus, giving continuity. When the food or the taste was unknown, it created a disconnection from the past and was not appreciated. The residents adjusted their lives according to the conditions of the nursing home. Nevertheless, the differences between “here” (in the nursing home) and “there” (in a private home) also tended to remind the residents that they now had become frail and dependent, resulting in sadness [4].

Mealtimes in nursing homes are social hubs and often the only time the residents meet each other. During the mealtimes, residents socialised with the other residents and/or staff. This has also shown to be an ideal that the staff at nursing homes promote, as an activity giving the opportunity to meet others and enable social interaction as part of the older persons’ rehabilitation [35, 36], although that is not always easy [15].

It is interesting that the residents seldom discussed the staff’s role during mealtimes. This can be due to the residents being relatively functional and thus not needing so much aid. However, when the staff was referred to, they seemed to be regarded in a role as a waiter for the residents, suggesting and serving food. Those residents who had the staff to choose the food were also satisfied with the staffs’ choices. Although the staff choosing the food may be regarded as paternalistic, our study suggests that letting others decide could also be an active choice of autonomy from the residents’ side.

Not being satisfied with the mealtime situation might develop into dissatisfaction with dignity and well-being. For example, Keller, Beck and Namasivayam [37] showed that food intake in nursing homes is influenced by the dining environment and social interactions during mealtimes. Environmental and social strategies should therefore be considered to encourage food intake. Implementation of nutritional guidelines, including meeting and atmosphere, has positive effects on experiences of mealtimes (Andersson et al: Putting staffs' beliefs about values of mealtime situations för long-term care residents' health and well-being into practice - a qualitative study, submitted). Therefore, to promote dignity and well-being by improving the mealtime environment, it is essential to include meeting and atmosphere, not only the physical environment [38]. It is apparent how the aspects of FAMM need to be taken into account when the mealtime situation is planned, as it is obvious how these aspects interact in the whole mealtime experience and give it meaning.

In many countries, the residents pay for their accommodation and meals in nursing homes. Therefore, it may be logical for some residents to view themselves as paying customers and thus prefer restaurant-style meals rather than family-style meals. The fact that the meals are served collectively can also be expected to have an impact on this impression. Other factors, such as the positive self-image of being served one’s meals in a restaurant manner, are probably also of importance. However, the view of residents in nursing homes as customers is problematic, as it may distract focus from the caring aspects of mealtimes in nursing homes. In a restaurant, you expect to have a choice of dishes and to be attended to by a service professional. Although there are commonalities with aspects of mealtime care, such a service approach might clash with staff’s self-image as trained caregivers and their view on what their care mission truly is. There might be a mistakenly degrading view among care professionals on mealtime service compared to caregiving. In our findings, we find evidence corresponding with the concept of person-centeredness as described by Feldthusen et al., namely, the residents wish for being unique, being heard and being an actor with shared responsibility [39].

Although not a primary finding in our analysis, it is worth mentioning that residents’ subjective voices are still not prominent in the research about their own mealtime experiences/perspectives in nursing homes, which was already concluded by Watkins et al. [15]. Thus, as paying guests and senior citizens, their voices should be taken into consideration when planning for meal services even in nursing homes.

## Methodological aspects

A wish in this review was to only include studies that had been published in journals publishing original peer-reviewed research articles. Although the measures taken in our search strategy, we might have missed relevant studies, which our manual search revealed. Our focus on research articles might have led to a miss of interesting results concerning residents ‘ experiences that can have been published in “grey” literature by governments, accreditation agencies or professional bodies. These results might have added an even broader picture of the residents’ experiences. However, by the systematic process we hope that the results cover the main issues about residents’ experiences and views of mealtime situations.

By using the JBI Critical Appraisal Tool in the quality appraisal and by deciding that at least 65% of the questions in the tool should be answered by yes the aim was to have a high level of quality in the included studies. Since 13 out of 16 studies were included there could be said that an overall appraisal was that studies had high or moderate degree of quality in this type of research. What was most often missing was a philosophical perspective in the methodological text and statements locating the researchers culturally or theoretically.

The results in this review come from different countries, and report varied organizations of mealtimes, which might not be easily translated to other situations. We have, however, tried to concentrate the results so that the main aspects, inherent different organizational aspects, can be seen as generalizable to different conditions. Still, transferability [40] can also be hard to achieve since the studies included are only from Western countries. Our search strategy was thorough, and it is therefore hard to explain why no studies turned up from other continents. Maybe research into this field needs more attention all over the world.

However, the main aspects shown in our study were relatively clear. The studies chosen to our review focused on different aspects of the mealtimes, where the organization of the mealtimes and the social aspects were most often in focus. This might give a bias for the results, as the other aspects of mealtimes are also important, and might have an impact on the satisfaction of the mealtimes as a whole. More studies are needed.

## Conclusion

This integrative review shows that residents in nursing homes want the possibility to choose both what to eat, with whom they will eat and where they eat. The mealtimes in nursing homes and how they are constructed have an important role in residents’ feelings of having control over life situations and can also strengthen residents’ identity, autonomy and continuity in life. Staff needs to be aware of all aspects of a mealtime situation to promote person-cantered care. Further research is needed to explore how different mealtime situations have an impact on nursing home residents’ lives as well as on the care the staff gives.

## Data Availability

The datasets used and/or analysed during the current study are available from the corresponding author upon reasonable request.

## Abbreviation

FAMM: Five Aspects Meal Model
